# Maternal and Paternal Genomes Differentially Affect Myofibre Characteristics and Muscle Weights of Bovine Fetuses at Midgestation

**DOI:** 10.1371/journal.pone.0053402

**Published:** 2013-01-14

**Authors:** Ruidong Xiang, Mani Ghanipoor-Samami, William H. Johns, Tanja Eindorf, David L. Rutley, Zbigniew A. Kruk, Carolyn J. Fitzsimmons, Dana A. Thomsen, Claire T. Roberts, Brian M. Burns, Gail I. Anderson, Paul L. Greenwood, Stefan Hiendleder

**Affiliations:** 1 J.S. Davies Non-Mendelian Genetics Group, School of Animal and Veterinary Sciences, Roseworthy Campus, The University of Adelaide, South Australia, Australia; 2 Robinson Institute, The University of Adelaide, South Australia, Australia; 3 NSW Department of Primary Industries, Beef Industry Centre, Trevenna Rd, University of New England, Armidale, New South Wales, Australia; 4 School of Paediatrics and Reproductive Health, The University of Adelaide, South Australia, Australia; 5 The University of Queensland, Centre for Animal Science, Queensland Alliance for Agriculture and Food Innovation, Rockhampton, Queensland, Australia; Universidad Europea de Madrid, Spain

## Abstract

Postnatal myofibre characteristics and muscle mass are largely determined during fetal development and may be significantly affected by epigenetic parent-of-origin effects. However, data on such effects in prenatal muscle development that could help understand unexplained variation in postnatal muscle traits are lacking. In a bovine model we studied effects of distinct maternal and paternal genomes, fetal sex, and non-genetic maternal effects on fetal myofibre characteristics and muscle mass. Data from 73 fetuses (Day153, 54% term) of four genetic groups with purebred and reciprocal cross Angus and Brahman genetics were analyzed using general linear models. Parental genomes explained the greatest proportion of variation in myofibre size of *Musculus semitendinosus* (80–96%) and in absolute and relative weights of *M. supraspinatus*, *M. longissimus dorsi*, *M. quadriceps femoris* and *M. semimembranosus* (82–89% and 56–93%, respectively). Paternal genome in interaction with maternal genome (*P*<0.05) explained most genetic variation in cross sectional area (CSA) of fast myotubes (68%), while maternal genome alone explained most genetic variation in CSA of fast myofibres (93%, *P<*0.01). Furthermore, maternal genome independently (*M. semimembranosus*, 88%, *P<*0.0001) or in combination (*M. supraspinatus*, 82%; *M. longissimus dorsi*, 93%; *M. quadriceps femoris*, 86%) with nested maternal weight effect (5–6%, *P<*0.05), was the predominant source of variation for absolute muscle weights. Effects of paternal genome on muscle mass decreased from thoracic to pelvic limb and accounted for all (*M. supraspinatus*, 97%, *P<*0.0001) or most (*M. longissimus dorsi*, 69%, *P<*0.0001; *M. quadriceps femoris*, 54%, *P<*0.001) genetic variation in relative weights. An interaction between maternal and paternal genomes (*P*<0.01) and effects of maternal weight (*P*<0.05) on expression of *H19*, a master regulator of an imprinted gene network, and negative correlations between *H19* expression and fetal muscle mass (*P<*0.001), suggested imprinted genes and miRNA interference as mechanisms for differential effects of maternal and paternal genomes on fetal muscle.

## Introduction

Skeletal muscle accounts for up to half of mammalian body mass [1] and has important functions in metabolic homeostasis [2], [3]. It is a major source of endocrine factors, including insulin-like growth factors -I (IGF1) and -II (IGF2), key components of the insulin-like growth factor (IGF) system and growth hormone – IGF axis, which are major regulators of pre- and postnatal muscle development and growth [4]–[7]. Skeletal muscle is composed of two major fibre types, type I (slow oxidative) fibres and type II (fast) fibres [2]. Myofibres originate from mesenchymal stem cells which differentiate into myoblasts during embryonic development [8]. Myoblasts fuse to form myotubes which develop into myofibres at the fetal stage [9]. In ruminants, myofibres differentiate during late fetal development into type I, type IIA (fast oxidative-glycolytic) and type IIX (fast glycolytic) myofibres [10], [11]. Thus, myofibre number is established during fetal development and postnatal skeletal muscle mass is largely determined prenatally [9], [12] by the interplay of a complex network of genetic and epigenetic factors [13]–[16].

Studies on postnatal muscle tissue of human, porcine and bovine revealed that genetics explained up to 45% of variation in slow myofibre percentage [17], up to 58% of variation in myofibre number [18] and 74% of variation in myofibre size [19], respectively. Similarly, using proxies such as lean body mass and lean tissue percentage, studies in human [20], [21] and porcine [18] demonstrated that genetics accounted for approximately 50–80% of variation in postnatal muscle mass. Apart from genetic factors that follow Mendelian rules of inheritance, prenatal muscle development and postnatal muscle phenotype may be affected by genetic and epigenetic factors with Non-Mendelian modes of inheritance. This includes effects of mitochondrial genome [22], X- and Y-chromosomes [23], [24], non-random X-inactivation [25], microRNA (miRNA) interference [26] and genomic imprinting [24], [27]–[29]. Genomic imprinting, i.e., parent-of-origin dependent allele-specific gene expression [30], has been described for genes with pivotal roles in myogenesis, including *IGF2* and its receptor *IGF2R* [31], [32]. In porcine, mapping and gene expression studies demonstrated that *IGF2* alleles explained up to 30% of variation in postnatal muscle mass [33]. The ovine callipyge (CLPG) mutation has provided an example of complex genetic and epigenetic effects on postnatal muscle phenotype. The CLPG mutation causes postnatal muscle hypertrophy only in heterozygous offspring and only when inherited through the paternal germline [34]. This polar overdominance changes imprinted gene expression, presumably by miRNA interference [35], and affects absolute and relative weights of specific muscles and muscle groups of the torso (e.g. *M. longissimus lumborum*) and pelvic limb (e.g. *M. semimembranosus, M. quadriceps femoris*), but not of the thoracic limb (e.g. *M. supraspinatus*) [36], [37]. The increased muscle mass of CLPG sheep is due to fast myofibre hypertrophy and results in higher glycolytic metabolism of affected muscles [38], [39]. A similar paternal polar overdominance effect on postnatal myofibre characteristics, muscle mass and growth has been described in porcine [40]. Furthermore, the ovine Carwell locus, which exerts paternal effects on weight of *M. longissimus dorsi* and a shift from type IIA to type IIX myofibres, was mapped to the same chromosome region as the CLPG mutation [41]–[43]. More recently, statistical modelling revealed significant parent-of-origin effects attributed to genomic imprinting on postnatal absolute and relative weights of specific muscles in porcine [27] and bovine [28].

Nutritional effects on prenatal myogenesis are well documented [12], [44]–[46], but data on parental genetic and epigenetic effects are lacking. To our knowledge, only one previous study investigated genetic effects on mammalian prenatal muscle. This report described significant individual sire effects on bovine fetal biceps weight in the last trimester of gestation [47]. However, the study was designed to test only for effects of different sires and did not address differential effects of maternal and paternal genomes. In the present study, we generated the largest fetal resource to date for the study of (epi)genetic effects on mammalian prenatal muscle development. This collection of defined bovine fetuses consists of both purebreds and reciprocal hybrids with Angus and Brahman genetics. The taurine (Angus) and indicine (Brahman) breeds are subspecies of the domestic cow, currently named *Bos taurus taurus* and *Bos taurus indicus*, respectively [48]. Both subspecies originated from the wild aurochs (*Bos primigenius*) and are commonly referred to as *Bos taurus* and *Bos indicus* (*Linnaeus*, *1758*; *Bojanus*, *1827*; loc. cit. http://www.itis.gov) [49]. This unique intra-species model with well defined divergent parental genomes allowed us to dissect maternal and paternal genome effects on fetal myofibre characteristics and absolute and relative muscle weights at midgestation (Day153, 54% term). We show, for the first time, significant differential effects of parental genomes, independently or in combination with non-genetic maternal effects, on specific fetal muscles. Furthermore, we correlated expression of the imprinted non-coding RNA H19, which harbors miRNAs and is involved in regulation of *IGF2* and *IGF1R*, with fetal muscle mass, demonstrating that imprinted genes and miRNA interference provide plausible mechanisms for observed differential effects of parental genomes on fetal muscle phenotype.

## Results

### Proportion of Variation Explained by Parental Genomes, Fetal Sex and Non-Genetic Effects

Myofibre characteristics determined in *M. semitendinosus* samples included number and cross-sectional area (CSA) of type I (slow) and type II (fast) myotubes and myofibres and total cell number and total cell CSA (**Figure S1**). Wet weights were determined for *M. supraspinatus*, *M. longissimus dorsi*, *M. quadriceps femoris* and *M. semimembranosus*. Since the four fetal groups with specific combinations of *Bos taurus taurus* (Bt) and *Bos taurus indicus* (Bi) genomes showed significant differences in carcass weights (**Figure S2**), relative muscle weights were analyzed in addition to absolute muscle weights to identify effects of parental genomes on muscle mass independent of fetal size.

**Figure 1:**
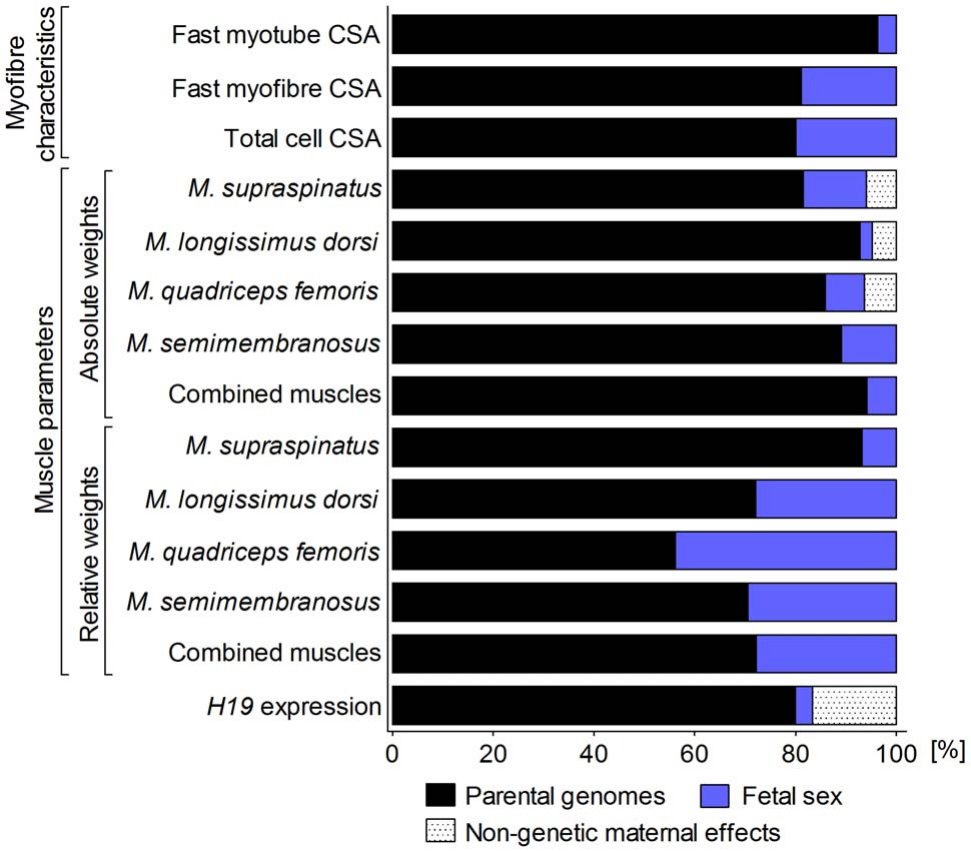
Relative contributions of parental genomes, fetal sex and non-genetic maternal effects to explained variation in fetal myofibre characteristics, absolute and relative muscle weights, and *H19* transcript abundance. Myofibre characteristics were determined in *M. semitendinosus.* Maternal and paternal genome, fetal sex and other significant effects were retained in the final general linear models as presented in **Table 1**. Non-genetic maternal effect: Final maternal weight at mid-gestation. CSA: Cross-sectional area. Total cell: All myofibres measured regardless of cell type. Combined muscle weights: Sum of *M. supraspinatus, M. longissimus dorsi*, *M. semimembranosus* and *M. quadriceps femoris* weight. Relative muscle weight: Absolute muscle weight divided by decapitated and eviscerated fetal carcass weight.

Significant final statistical models for studied muscle parameters with adjusted *R* ^2^ values and significance levels of retained variables are presented in **Table 1**. Parental genomes, fetal sex, and effects of maternal weight, caused by non-genetic variation and nested within maternal genomes (see methods), each contributed differentially to muscle parameters (**Figure 1**). Parental genome was the most important source of variation for all studied traits with significant final statistical models. Maternal and paternal genomes together explained most of the variation in myofibre size (80–96%), absolute muscle weights (82–89%) and relative muscle weights (56–93%). Fetal sex contributed less to variation in myofibre characteristics (4–20%) and absolute (2–13%) and relative muscle weights (7–44%). Non-genetic maternal effects of final maternal weight accounted for some variation in absolute weights of *M. supraspinatus*, *M. longissimus dorsi* and *M. quadriceps femoris* (5–6%). Combined absolute and relative muscle weight showed parental genome contributions of 94% and 72%, respectively (**Figure 1**).

**Table 1:** Summary of the final general models (type III sums of squares) for myofibre characteristics, muscle weight parameters and H19 gene expression with adjusted *R* ^2^ values and significance levels (*P-*values) of models and variables.

		*P*-values					
Myofibre characteristics	*R* ^2^	Model	Maternal genome	Paternal genome	Fetal sex	Maternal×Paternal genome^b^	Final maternal weight (Maternal genome)^c^
Fast myotube CSA^a^	0.152	0.0043	ND	ND	0.4337	0.0129	
Fast myofibre CSA^a^	0.111	0.0117	0.0031	0.7345	0.1390		
Total cell CSA^a^	0.101	0.0160	0.0076	0.4280	0.1434		
**Absolute muscle weights**							
*M. supraspinatus*	0.689	8.7E-17	ND	2.3E-07	7.0E-04		0.0112
*M. longissimus dorsi*	0.649	1.2E-15	ND	6.9E-08	0.2828		0.0420
*M. quadriceps femoris*	0.666	1.0E-14	ND	2.1E-05	0.0457		0.0256
*M. semimenbranosus*	0.595	7.2E-12	5.1E-12	0.04974	0.0026		
Combined muscles	0.667	2.9E-14	5.0E-13	3.3E-05	0.0095		
**Relative muscle weights**							
*M. supraspinatus*	0.210	3.3E-04	0.5294	2.7E-05	0.2327		
*M. longissimus dorsi*	0.441	4.8E-09	0.0014	9.8E-08	1.6E-04		
*M. quadriceps femoris*	0.332	1.6E-06	0.0048	1.2E-04	1.4E-04		
*M. semimenbranosus*	0.136	0.0115	0.0176	0.4209	0.0637		
Combined muscles	0.517	2.1E-09	2.3E-04	2.2E-06	5.9E-06		
***H19*** ** expression**	0.350	4.0E-06	ND	ND	0.1288	0.0051	0.0296

Only *P* **-**values for factors, interactions and nested effects retained in the final model are shown.

a Total cell CSA: Average cross-sectional area of muscle cells irrespective of cell type.

b Maternal×paternal genome: Effect of maternal and paternal genome interaction.

c Final maternal weight (maternal genome): Effect of final maternal weight nested in maternal genome. ND: Not determined because of significant interaction and/or nested effect of final maternal weight.

**Figure 2:**
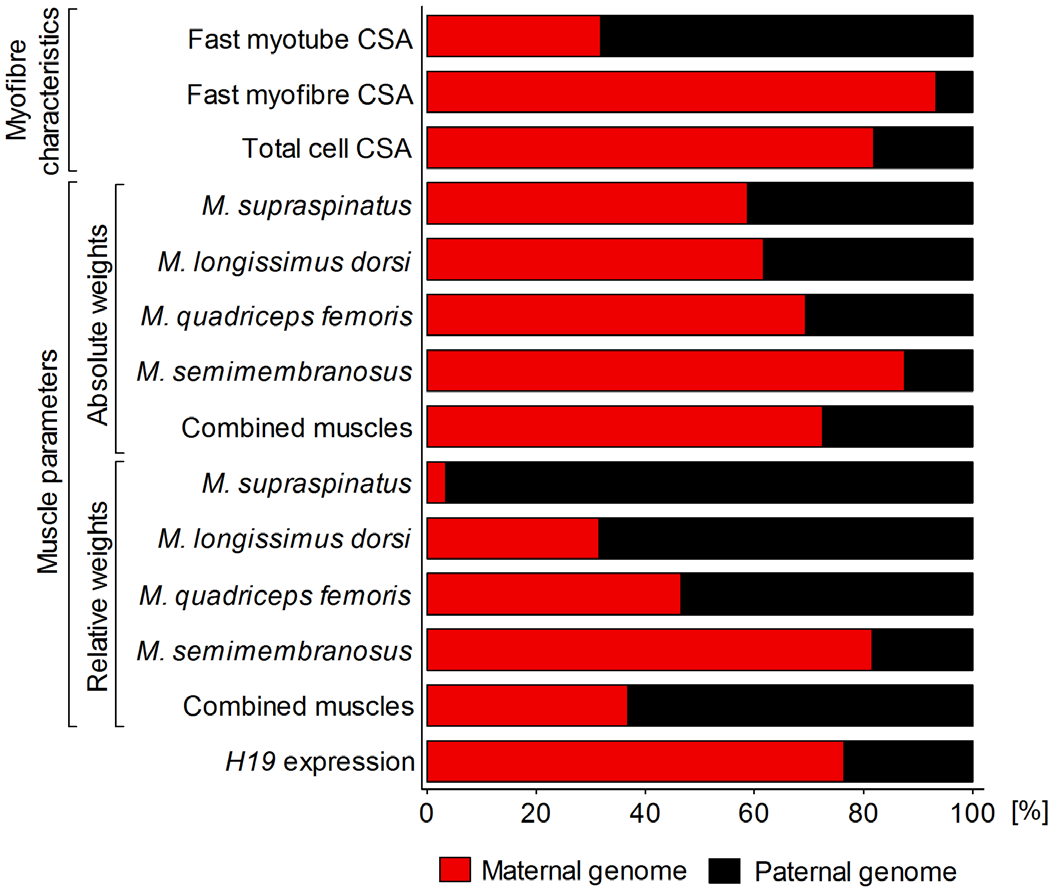
Relative contributions of maternal and paternal genome to genetic variation in fetal myofibre characteristics, absolute and relative muscle weights, and *H19* transcript abundance. Myofibre characteristics were determined in *M. semitendinosus.* CSA: Cross-sectional area. Total cell: All myofibres measured regardless of cell type. Combined muscle weights: Sum of *M. supraspinatus, M. longissimus dorsi*, *M. semimembranosus* and *M. quadriceps femoris* weight. Relative muscle weight: Absolute muscle weight divided by decapitated and eviscerated fetal carcass weight.

The relative contributions of maternal and paternal genomes to total explained (epi)genetic variation in myofibre size and muscle weights are shown in **Figure** **2**. Maternal genome explained most of the (epi)genetic variation in fast myofibre CSA (93%) whereas the paternal genome accounted for most of the variation in fast myotube CSA (68%). Maternal genome again explained most of the variation in total cell CSA (82%). Maternal genome also explained most of the genetic variation (59–88%) in all absolute muscle weights. Paternal genome, in contrast, explained most of the genetic variation (54–97%) in relative weights of *M. supraspinatus*, *M. longissimus dorsi* and *M. quadriceps femoris*. However, maternal genome accounted for 82% of genetic variation in relative weight of *M. semimembranosus.* Combined absolute muscle weight was predominantly affected by maternal genome (73%) while combined relative muscle weight showed a stronger effect of paternal genome (63%). Overall, the data clearly showed a distinct pattern of effects of maternal and paternal genomes with an increase of maternal genome contributions (or conversely, a decrease of paternal genome contributions) to variation in absolute and relative weights of muscles from the thoracic limb (*M. supraspinatus*) to muscles from the torso (*M. longissimus dorsi*) and pelvic limb (*M. quadriceps femoris* and *M. semimembranosus*) (**Figure** **2**).

### Specific Effects of Bt and Bi Genomes, Fetal Sex and Maternal Weight

**Figure 3:**
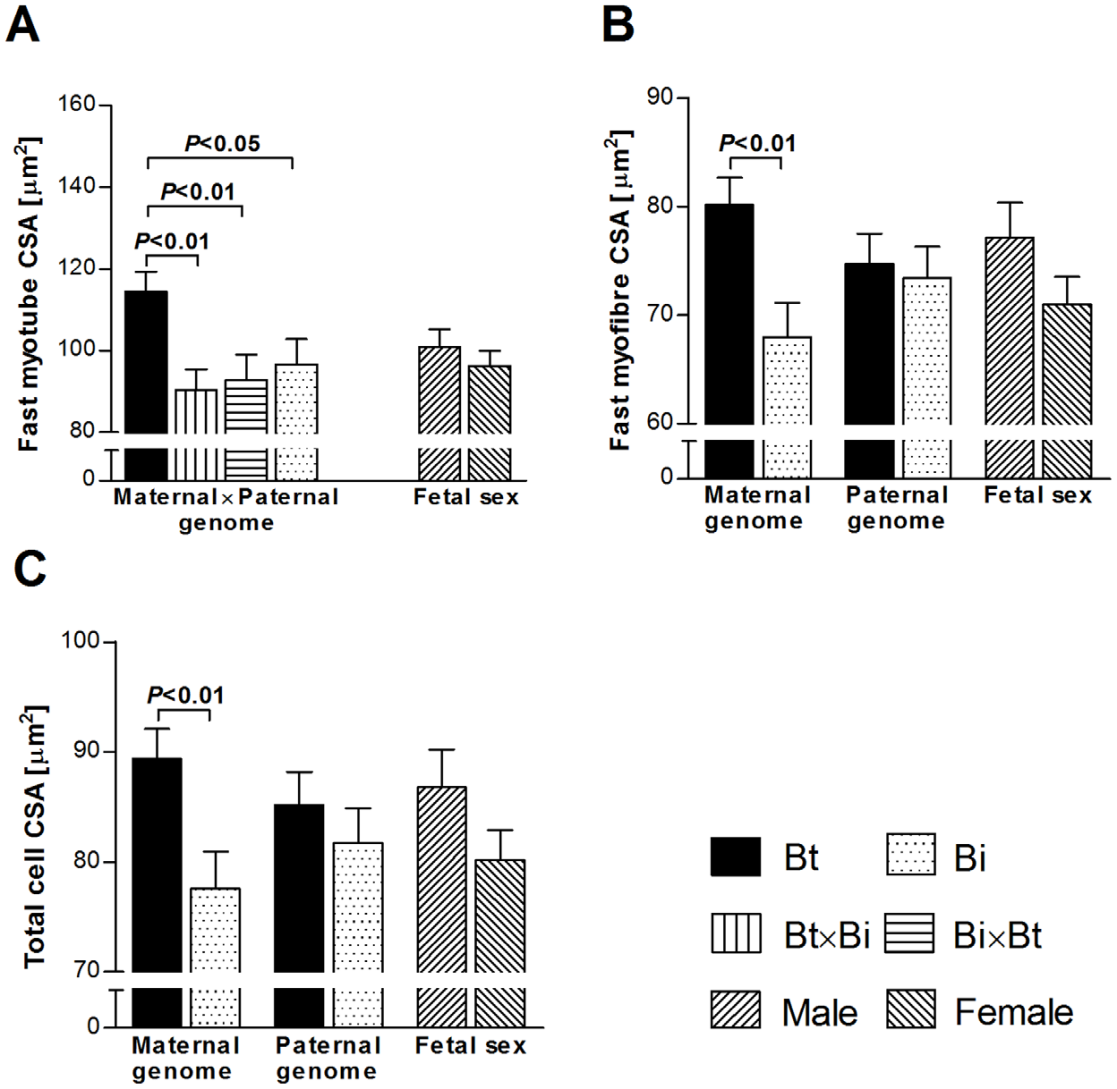
Specific effects of maternal genomes, paternal genomes and fetal sex on fetal myofibre characteristics of *M. semitendinosus* at midgestation. Least square means with standard errors of means are shown and *P*-values for significant differences (*t*-test) between means for fast myotube CSA (**A**), fast myofibre CSA (**B**) and total cell CSA (**C**) are indicated. CSA: Cross-sectional area. Total cell: All myofibres measured regardless of cell type. Bt: *Bos taurus taurus*, Angus. Bi: *Bos taurus indicus*, Brahman.

Least square means for specific effects of *Bos taurus taurus* (Bt, Angus) and *B. taurus indicus* (Bi, Brahman) maternal and paternal genomes, fetal sex and non-genetic maternal effects of final maternal weight, as detailed in statistical models for myofibre characteristics and muscle weights (**Table 1**), are presented in **Figures 3** **,** **4** **,** **5** **, and** **6**. Fast myotube CSA was affected by a significant interaction between maternal and paternal genomes (*P<*0.05). Fetuses with Bt×Bt genomes had larger CSA (*P<*0.05–0.01) than fetuses of other genetic combinations (**Figure 3A**). Maternal genome significantly affected fast myofibre CSA and total cell CSA (both *P<*0.01) with Bt genomes causing larger CSA than Bi genomes (**Figure 3B,C**).

**Figure 4:**
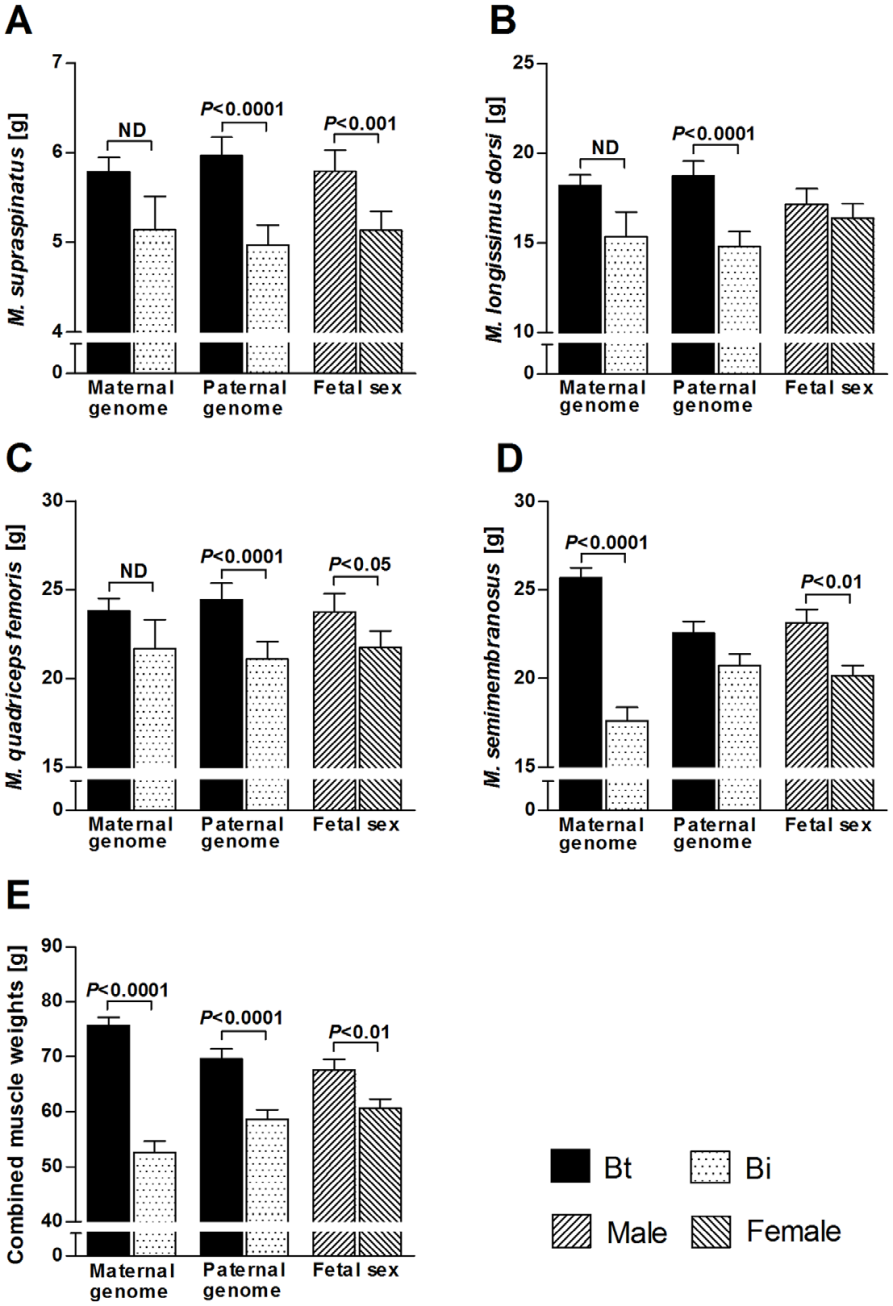
Specific effects of maternal genomes, paternal genomes and fetal sex on fetal absolute muscle weights at midgestation. Least square means with standard errors of means are shown and *P*-values for significant differences (*t*-test) between means for *M. supraspinatus* (**A**), *M. longissimus dorsi* (**B**), *M. quadriceps femoris* (**C**), *M. semimembranosus* (**D**) and combined muscle weight (sum of weights of dissected muscles) (**E**) are indicated. ND: Not determined because of significant nested effect of final maternal weight (see **Figure 5**). Bt: *Bos taurus taurus*, Angus. Bi: *Bos taurus indicus*, Brahman.

**Figure 5:**
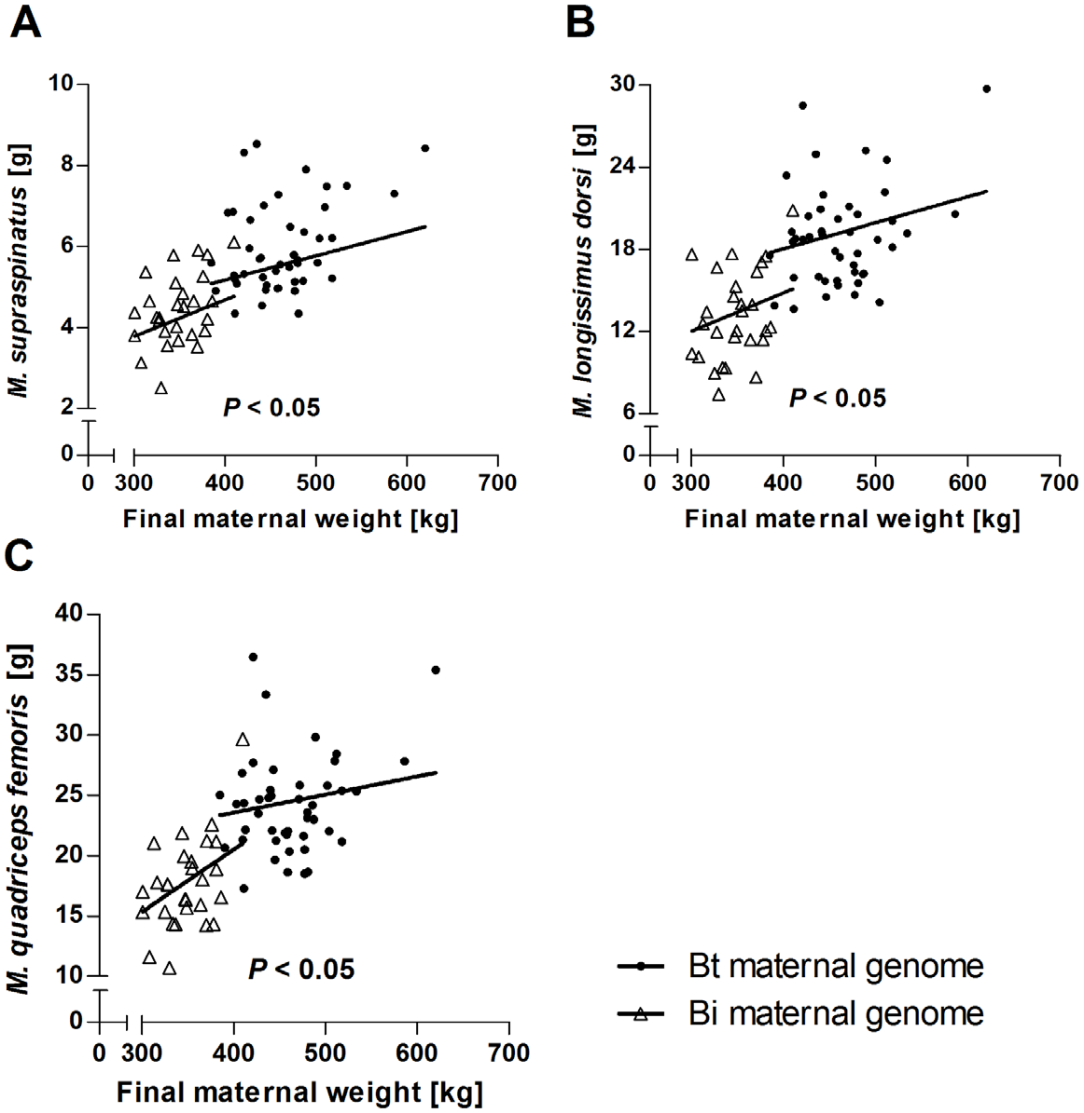
Effects of final maternal weight nested within maternal genomes on fetal absolute muscle weights at midgestation. *P*-values (ANOVA) of significant linear regressions within Bt and Bi maternal genetics on absolute weights of *M. supraspinatus* (**A**), *M. longissimus dorsi* (**B**) and *M. quadriceps femoris* (**C**) are indicated. Bt: *Bos taurus taurus*, Angus. Bi: *Bos taurus indicus*, Brahman.

**Figure 6:**
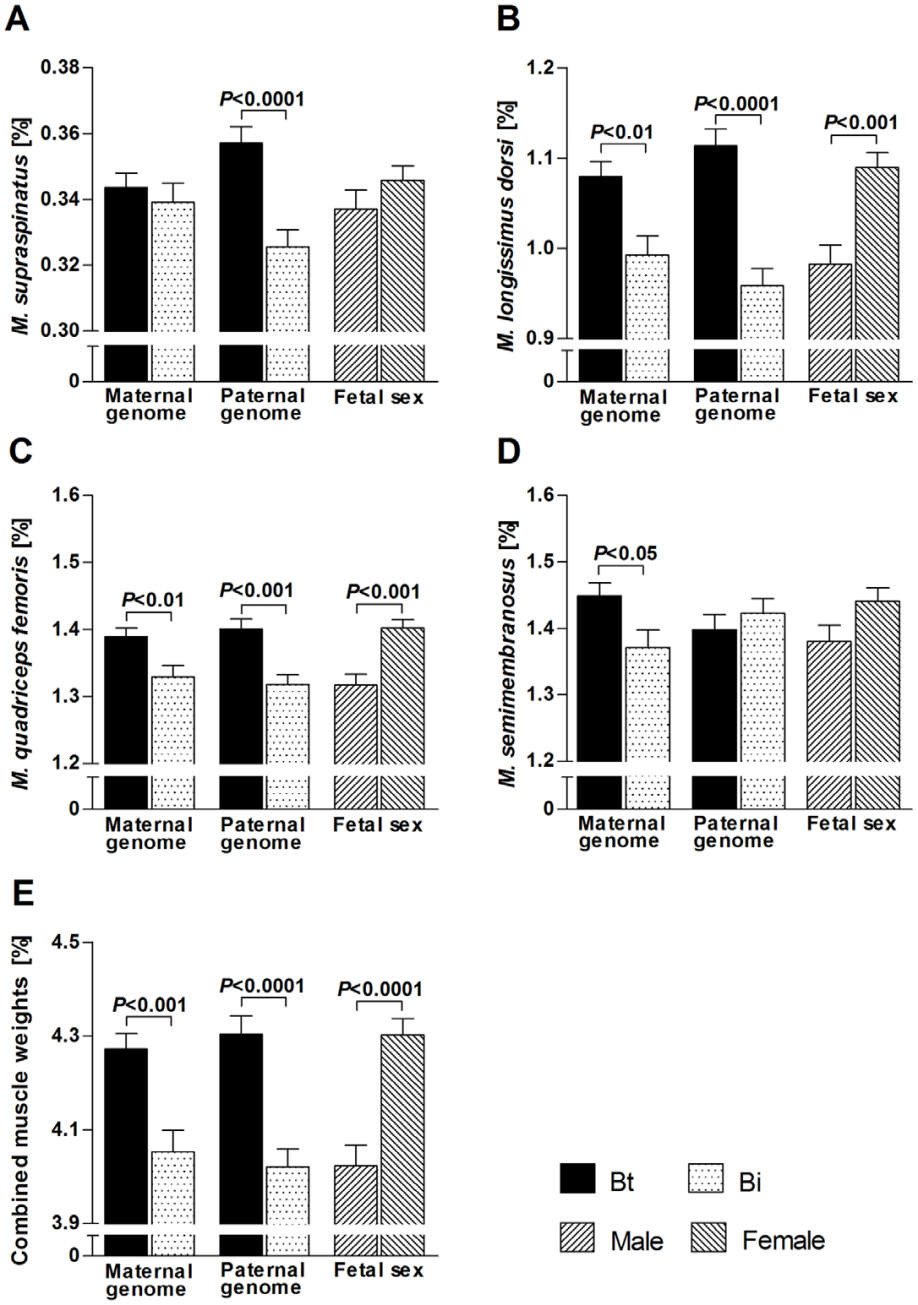
Specific effects of maternal genomes, paternal genomes and fetal sex on fetal relative muscle weights at midgestation. Relative muscle weights were calculated as absolute muscle weight divided by fetal carcass weight. Least square means with standard errors of means and *P*-values for significant differences (*t*-test) between means for *M. supraspinatus* (**A**), *M. longissimus dorsi* (**B**), *M. quadriceps femoris* (**C**) and *M. semimembranosus* (**D**) are indicated. Combined relative muscle weight is the sum of relative weights of dissected muscles. Bt: *Bos taurus taurus*, Angus. Bi: *Bos taurus indicus*, Brahman.

Maternal genome significantly affected absolute weights of all muscles (**Figure 4A–D**), but *M. supraspinatus, M. longissimus dorsi* and *M. quadriceps femoris* also showed significant non-genetic effects of final maternal weight nested within maternal genome (all *P*<0.05, see below). Maternal genome effects, independent of maternal weight, were detected for *M. semimembranosus* (*P<*0.0001). Paternal genome, in contrast, independently and strongly affected absolute weights of *M. supraspinatus, M. longissimus dorsi* and *M. quadriceps femoris* (all *P<*0.0001), but not *M. semimembranosus*, a muscle strongly affected by maternal genome (see above). Combined muscle weights showed significant effects of maternal and paternal genome that were stronger for the maternal genome. Irrespective of maternal or paternal origin Bt genome always increased, and Bi genome always decreased, absolute muscle weights. Fetal sex significantly affected absolute weights of *M. supraspinatus* (*P*<0.001), *M. quadriceps femoris* (*P*<0.05) and *M. semimembranosus* (*P*<0.01) with heavier muscles in males than in females (**Figure 4A,C,D**). Non-genetic effects of final maternal weight, nested within maternal genome, on absolute weights of *M. supraspinatus, M. longissimus dorsi* and *M. quadriceps femoris* (*P*<0.05) indicated positive linear relationships for Bi and Bt, but with a higher intercept and less slope in Bt (**Figure 5A–C**). Only one of the quadratic maternal weight effects tested yielded a significant result (*M. quadriceps femoris, P*<0.01). Examination of plotted curves with individual data points revealed that this was dependent upon two heavy dams with high leverage (see methods and **Figure S3**). Therefore, we fitted linear effects throughout. Nested effects of post conception maternal daily weight gain were not significant for any of the investigated muscle parameters.

Maternal genome had moderate effects on relative weights of *M. longissimus dorsi* (*P*<0.01), *M. quadriceps femoris* (*P<*0.01) and *M. semimembranosus* (*P*<0.05), but not *M. supraspinatus*. Paternal genome showed strong effects on *M. supraspinatus* (*P<*0.0001), *M. longissimus dorsi* (*P<*0.0001) and *M. quadriceps femoris* (*P<*0.001), but not *M. semimembranosus*. Combined relative muscle weight showed stronger effects of the paternal genome. Again, as for absolute muscle weights, Bt genome increased relative muscle weights irrespective of parental origin (**Figure 6A–D**). Strong fetal sex effects were present for relative weights of *M. longissimus dorsi* (*P<*0.001) and *M. quadriceps femoris* (*P<*0.001), with greater weights in females than in males (**Figure 6B,C**).

### Expression of the H19 lincRNA

**Figure 7:**
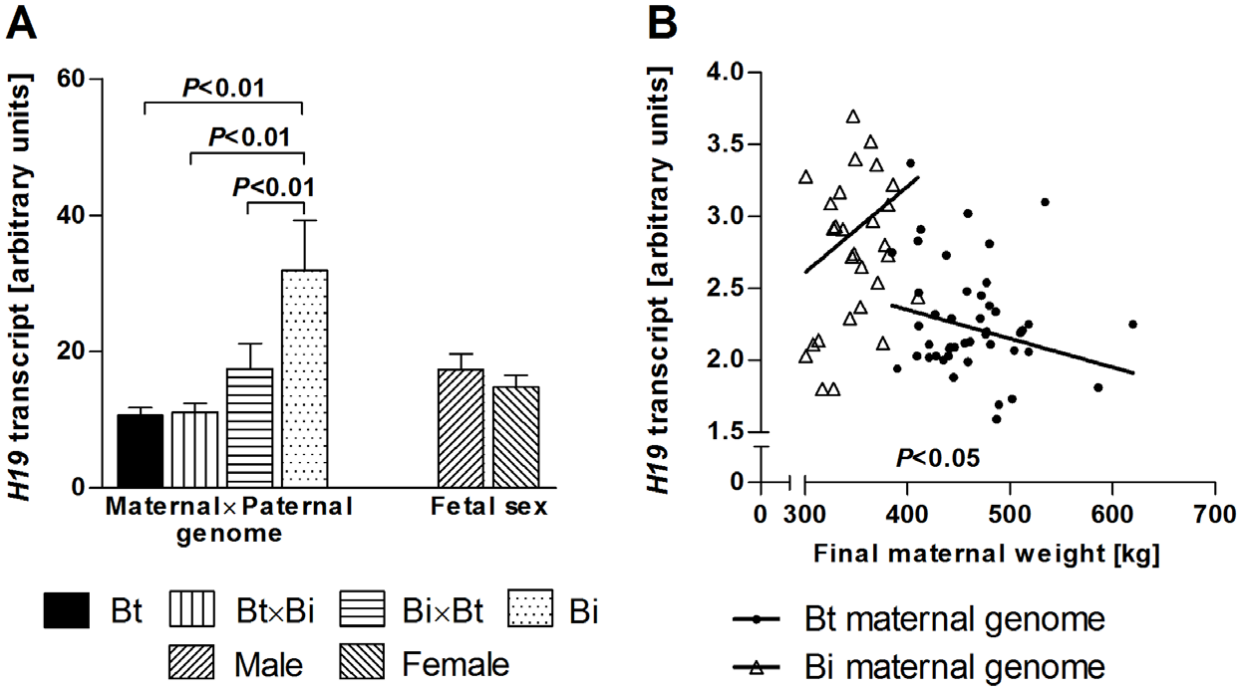
Effects of interaction of maternal and paternal genomes, fetal sex and final maternal weight nested within maternal genetics on *H19* transcript abundance in fetal *M. semitendinosus* at midgestation. Least square means with standard error of means and *P*-values for significant differences (*t*-test) between means (**A**) and significant regressions of final maternal weight nested within Bt and Bi maternal genomes (**B**) are shown. Bt: *Bos taurus taurus*, Angus. Bi: *Bos taurus indicus*, Brahman.

**Figure 8:**
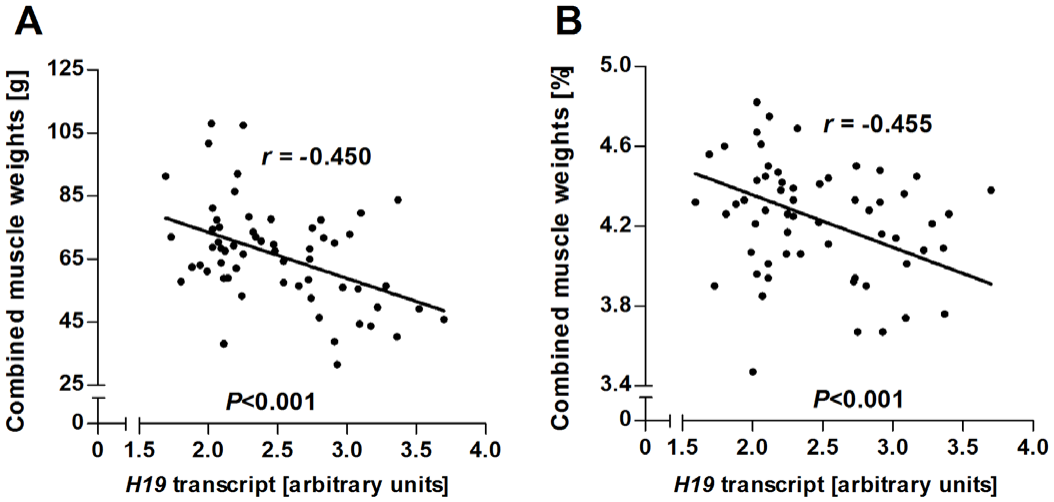
Regressions of fetal muscle mass at midgestation on *H19* transcript abundance. (**A**) Absolute muscle mass and (**B**) relative muscle mass. Muscle mass is combined absolute and relative weights of *M. supraspinatus*, *M. longissimus dorsi*, *M. quadriceps femoris* and *M. semimembranosus. P*-values and Pearson correlation coefficients (*r*) are indicated.

Expression of the H19 large intergenic non-coding RNA (lincRNA) was measured by real-time quantitative PCR in *M. semitendinosus* samples. Transcript abundance was significantly affected by an interaction between maternal and paternal genomes (*P<*0.01) (**Table 1**). Fetuses with Bi×Bi genome showed higher levels of *H19* transcript (*P<*0.01) than fetuses of other genetic combinations (**Figure 7A**). Transcript abundance was also affected by final maternal weight (*P<*0.05) nested within maternal genome (**Figure 7B**). Subsequent regression analyses revealed significant negative relationships (*P*<0.001) between *H19* transcript abundance and combined absolute and relative muscle weight (**Figure 8A,B**).

## Discussion

To our knowledge, this is the first study to examine effects of maternal and paternal genome on fetal myofibre characteristics and muscle mass. Our results showed that differential effects of parental genomes were the most important determinants of fetal muscle phenotype at midgestation. Fetal sex and non-genetic effects of final maternal weight had a significant but lesser impact on some investigated muscle parameters (**Figure 1**). Considering the fetal programming of skeletal muscle development [9], [12], these findings are consistent with generally medium to high heritabilities reported for postnatal myofibre size and muscle mass in mammals, including bovine [18], [19], [24], [50], [51]. Since myotubes are immature myofibres that decrease in size as myogenesis progresses [52], both the predominant contribution of the paternal genome to variation in fast myotube cross sectional area (CSA), and the predominant contribution of the maternal genome to variation in fast myofibre CSA (**Figure 2**), indicate specific roles of maternal and paternal genomes in myofibre differentiation and maturation.

The observed differences between *Bos taurus taurus* (Bt) and *Bos taurus indicus* (Bi) genomes likely result from allelic differences in genes with parent-of-origin effects controlling myofibre development. Evidence for subspecies differences in postnatal fibre type ratios and size, and in absolute postnatal muscle weights of Bt and Bi breeds has been reported previously [53]–[55]. Differential parental effects were masked in total cell CSA, which was predominantly affected by maternal genome (**Figure 2**). Muscle specific differences in fibre type composition and size [56] could explain some of the varying contributions of maternal and paternal genomes to different muscles. The present data suggest that maternal genes are important determinants of myofibre development and muscle mass.

Variation in the maternally inherited mitochondrial genome has been associated with effects on postnatal muscle mass [22], but specific effects of maternal genes in myogenesis remain, to our knowledge, unexplored. The present results are in agreement with recent data obtained by statistical modelling and imprinted quantitative trait loci (QTL) analyses which suggested significant maternal parent-of-origin effects for postnatal muscle traits [27]–[29]. In contrast, paternally expressed genes with effects on myogenesis have been identified previously and were studied in detail. This includes the imprinted Delta-like 1 homolog (*DLK1*), which has been implicated in the commitment and/or proliferation of fetal myoblasts [39] and in increased postnatal myofibre diameter and muscle mass [39], [57]. Further examples of gene-specific genetic and epigenetic regulatory mechanisms that could explain effects of maternal and paternal genomes on fetal muscle phenotype observed in the present study are found in the IGF1-AKT/PKB pathway [58]. In the mouse embryo, paternally expressed IGF2 is required for fibre type specification [59]. This imprinted gene has been identified as a QTL for postnatal muscle mass [31], [60] and encodes a miRNA in intron 2 that targets transcripts of the non-imprinted *IGF1* gene [61]. Several other genes in this pathway, including *PTEN*, a gatekeeper for the accretion of muscle mass [7], are also targeted by miRNAs [13], [62]. The significance of allelic differences in miRNA target sequences for regulation of muscle mass by epistatic miRNA interference has been demonstrated with myostatin alleles in the ovine model [26]. Genome sequences of *Bos taurus taurus* and *Bos taurus indicus* revealed genomic variation [48], [63] that provides a basis for maternal and paternal (epi)genetic effects on myogenesis described in the present study.

The imprinted long intergenic non-coding (linc) RNA H19 is maternally expressed at high levels in embryonic and fetal tissues, including skeletal muscle [64], [65]. The H19 gene is located immediately downstream of *IGF2* and involved in regulation of *IGF2* expression. More recently, *H19* has been identified as the master regulator of an imprinted gene network with important roles in growth and development [66]. The *H19* transcript was further shown to harbor a miRNA that suppresses *IGF1R* expression and prenatal growth [67], [68]. Gene expression data generated in the present study demonstrated significant differences in *H19* transcript abundance of *M. semitendinosus* from fetuses with different parental combinations of Bt and Bi genomes (**Figure 7**). In human, *H19* expression is also affected by genetic background [69]. Furthermore, *H19* expression was significantly negatively correlated with absolute and relative fetal muscle mass (**Figure 8**). This is consistent with the previously reported role of *H19* as a negative regulator of prenatal growth and development [68]. Thus, imprinted gene expression and miRNA interference are plausible mechanisms for differential effects of maternal and paternal genomes observed in the present study.

Our data indicated predominant contributions of the maternal genome to variation in absolute fetal muscle weights and predominant contributions of the paternal genome to variation in relative fetal muscle weights (**Figure 2**). With respect to maternal genome, these results are in agreement with data available from an analysis of parent-of-origin effects on postnatal bovine muscle, where absolute muscle weights were predominantly affected by imprinted maternal genetic factors [28]. The genetic conflict hypothesis of genomic imprinting states that paternally expressed genes promote, and maternally expressed genes limit, fetal growth [70]. Accordingly, maternal genes are expected to control fetal size to avoid detrimental effects for the mother that are associated with higher nutrient transfer to the fetus and increased birthweight [70]. In the present study, fetuses with different maternal and paternal combinations of Bt and Bi genomes showed significant differences in carcass weight (**Figure S2**) that are consistent with a phenotypic pattern of genomic imprinting for maternally expressed genes (see Figure 1 in [71]) affecting fetal size. Correlations between absolute muscle weights and fetal carcass weight ranged from r = 0.88 (*M. longissimus dorsi*, *P<*0.0001) to r = 0.95 (*M. quadriceps femoris*, *P<*0.0001). Effects of the maternal genome on absolute muscle weights are, therefore, likely to be primarily correlated effects of maternal (epi)genetics on fetal size, presumably via imprinted genes [70], [71] and/or epistatic interaction of miRNAs and their target sites (see above). However, mitochondrial DNA [22], [72], or X-chromosome effects [23], [25] could also contribute to Bt and Bi maternal (epi)genetic effects on muscle phenotype (**Figure 3** **,** **4**).

Predominance of parental genomic contributions to muscle weights varied from maternal for absolute weights to paternal for relative weights. An exception was *M. semimembranosus,* which showed only a weak maternal (*P*<0.05) and no paternal genome effect (**Figure 2** **,** **4** **,** **6**). Considering the genetic conflict hypothesis [70], it appears that the full extent of paternal genome effects on muscle mass and shape should manifest postnatally, without causing detrimental effects to mother or fetus at parturition. Such effects could nevertheless be expected to be programmed prenatally [9], [12] and to be independent of absolute fetal muscle weights. This interpretation is consistent with the imprinting status of major regulators of fetal muscle development and growth in bovine e.g. paternally expressed growth promoting *IGF2* and maternally expressed growth inhibiting *IGF2R* [73], [74]. Imprinted gene effects with paternal mode of expression responsible for increased muscle mass in ovine (*DLK1*) and porcine (*IGF2*) manifest postnatally [31], [41], [57], [60].

Analyses of the proportion of parental contributions to muscle traits revealed that contributions of the maternal genome to absolute and relative fetal muscle mass increased (or conversely, contributions of the paternal genome decreased) from thoracic limb to torso and pelvic limb. This novel spatial effect of the maternal genome mirrored paternal effects on muscle mass observed in sheep with the polar overdominant callipyge mutation [34], [36], [37]. Consistent with our findings, a recent study in porcine identified a quantitative trait locus (QTL) with maternal polar overdominance that affected postnatal pelvic limb muscle mass [29]. Moreover, statistical modelling of parent-of-origin effects on postnatal muscle mass in porcine and bovine also showed a preponderance of maternal effects attributed to genomic imprinting [27], [28]. The significant switch in gene expression, including imprinted transcripts from the *DLK1-DIO3* region, in ovine *M. longissimus dorsi* from fetus to neonate [75], could indicate developmental stage specific roles of maternal and paternal genomes in myogenesis. Interestingly, the imprinting status of genes can change from monoallelic to non-imprinted biallelic expression during development [76]–[78]. Statistical analyses of experimental data for postnatal growth and development in mouse identified multiple imprinted QTL with complex temporal patterns of parent-of-origin effects [71]. It is tempting to speculate that such effects could also be spatial.

Significant effects of sex on postnatal muscle mass of mammals, including bovine, have been reported [18], [79]–[81], but the present study is the first to examine sex effects in prenatal myogenesis. In agreement with fetal programming of postnatal muscle mass discussed above (see maternal and paternal genomes), sex explained greater proportions of variation in relative fetal muscle weights than in absolute muscle weights (**Figure 1**). Male fetuses had higher absolute muscle weights but lower relative muscle weights than females (**Figure 4** **,** **6**). The latter findings are in agreement with results for postnatal muscle weights in porcine [79] and ovine [82]. In the present study, fetal sex had no effect on relative weight of *M. supraspinatus*, a shoulder muscle, but significantly affected the relative weights of *M. longissimus dorsi* (loin) and *M. quadriceps femoris* (pelvic limb) (**Figure 6**). This is again similar to results obtained for postnatal muscle mass in ovine [82], where sex had no effect on shoulder muscle percentage but significantly affected loin muscle percentage, with greater muscle percentage in females than in males. An explanation for these results could be that fetal shoulder muscle mass is under strong selection because of its relevance for birthing difficulties and thus survival. The loin and pelvic limb region of females may require a higher relative muscle weight to maintain sex-specific postnatal proportions and reproductive functions, which may be programmed during fetal development.

Our analyses identified significant contributions of final maternal weight (FMW) to variation in absolute fetal muscle weights and *H19* expression at midgestation (**Figure 1**). These non-genetic maternal effects were estimated as nested effects within maternal genetics using type I sums of squares in the final linear models, allowing the removal of maternal genetic contributions from effects of FMW (see methods). Non-genetic maternal components can be explained by differences in environmental factors acting on dams before they were recruited for the experiment. These environmental effects could not be erased during several weeks of adjustment under a controlled environment prior to the start of the experiment. To our knowledge, pre-conception non-genetic maternal contributions to variation in fetal muscle mass have not been reported previously. The estimated regression coefficients suggested that the same mechanisms affect fetal muscle mass in dams with Bt and Bi genomes (**Figure 5** **,** **7**).

In conclusion, we have shown for the first time, that fetal muscle development is differentially affected by maternal and paternal genome, independently, or in combination with non-genetic maternal effects. Our statistical analyses of effects of parental genomes, and molecular data for the imprinted maternally expressed lincRNA H19, suggested that imprinted gene networks [66] and epistatic miRNA interference [26] could be major drivers of the observed parental effects on fetal muscle traits. Our conclusions are supported by results from statistical modelling of postnatal muscle traits [24], [27], [28] which identified parent-of-origin effects attributed to imprinted genes as a major source of variation. Detailed molecular profiles are now required to elucidate genetic, epigenetic and non-genetic components and interactions that control variation in prenatal muscle traits. Our data further suggest that specific combinations of (epi)genetic and non-genetic factors can be used to optimise fetal, and therefore, postnatal muscle development and phenotype. Non-Mendelian (epi)genetic and non-genetic maternal effects can help understand unexplained variation in postnatal muscle traits. These traits may be highly variable within populations, even when genetics and environment are well controlled [83], [84].

## Materials and Methods

### Cattle and Fetuses

All animal experiments and procedures described in this study were approved by The University of Adelaide Animal Ethics Committee (No. S-094-2005 and S-094-2005A). We used animals and semen of the Angus and Brahman breeds to study differential parental genome effects on fetal muscle phenotype at midgestation. The two breeds are subspecies of domestic cow, commonly referred to as *Bos taurus* and *Bos indicus*, respectively [48], [49]. Nulliparous Angus and Brahman dams which were approximately 16–20 months of age were purchased from farms in South Australia and Queensland and transferred to, and maintained at, Struan Agricultural Centre, South Australia. Animals were on pasture supplemented by silage. After an adjustment period of 3–4 weeks the animals received standard commercial estrous cycle synchronization as described previously [85]. All fetuses were sired by two Brahman and three Angus bulls. Dams were pregnancy tested by ultrasound scanning and fetuses recovered in an abattoir at Day 153±1 of gestation. Fetuses were removed from the uterus, eviscerated, vacuum packed and stored frozen at −20°C until further processing. Final maternal weight (FMW) was recorded and average maternal daily weight gain (MDG) was calculated as FMW minus weight at conception divided by gestation length (**Figure S4**). We analyzed 73 fetuses in total, including 23 Bt×Bt, 22 Bi×Bt, 13 Bt×Bi and 15 Bi×Bi (paternal genetics listed first) with both sexes represented in each genetic group. The distribution of Bt and Bi maternal and paternal genomes, and of females and males, are shown in **Table S1.**

### Muscle Dissection and Weights

Fetuses were thawed and the head removed by disarticulation between the *Os occipitale* and first cervical vertebra atlas. *Musculus supraspinatus, M. longissimus dorsi*, *M. semimembranosus* and *M. quadriceps femoris* (consisting of *M. rectus femoris, M. vastus medialis, M. vastus intermedius* and *M. vastus lateralis*) were dissected from both sides of the fetus. *M. longissimus dorsi* was defined from the 7^th^ rib to the natural caudal end of the muscle, at the apophysis of the lumbosacral. The dissection protocol was based on Budras and Habel [86] and muscle nomenclature according to Tucker [87]. *M. semimembranosus* was obtained from 61 fetuses due to damage to some specimens from sampling adjacent *M. semitendinosus* for immunohistochemistry, described below. Dissected muscles from both sides of the fetus were weighed and absolute muscle weight was recorded as the mean weight for each muscle. Combined muscle weights were calculated as the sum of mean weight of each dissected muscle. Relative muscle weights, reflecting fetal muscle proportions, were calculated as muscle weight divided by the weight of the decapitated eviscerated fetus (see **Figure S2**).

### Muscle Immunohistochemistry

At the time of fetus collection, a section of *M. semitendinosus* was cut from the centre of the muscle and mounted using gum tragacanth (Sigma Chemical Company, St. Louis, MO; prepared 5% wt/vol in distilled, deionized H_2_O) onto a cork block, with muscle fibres running perpendicular to the cork block. Samples were frozen by immersion in iso-pentane cooled to approximately −160°C in liquid nitrogen, before storage at −80°C. Muscle tissue preparation and immunohistochemical staining followed the protocol by Greenwood et al. [11]. Briefly, 10-µm-thick, serial cross-sections were cut from each frozen sample using a cryostat microtome (ThermoShandon AS 620 Cryostat SME, Thermotrace Ltd., Noble Park, Victoria, Australia). After air-drying, cross-sections were stained against type I (slow) (clone WBMHC, Novocastra, Newcastle upon Tyne, UK; diluted 1∶100 in PBS) and type II (fast) (clone MY-32, Sigma; diluted 1∶400 in PBS) myosin heavy chain isoforms. Staining using these antibodies was previously shown to discern these myofibre types in ruminant fetal muscle [46]. They were revalidated in bovine fetal muscle using myofibrillar ATPase staining for the present experiment. The stained sections were dehydrated and cleared using graded ethanols and xylenes to produce slides using a xylene-based mounting medium.

### Myofibre Classification and Morphometry

Microscopic image analysis was used to classify and measure myofibres on stained slides. A Zeiss AxioPlan2 microscope fitted with Plan-Neofluar objectives (Carl Zeiss Pty. Ltd., Goettingen, Germany) and a Fujix colour digital camera (FUJIFILM Australia Pty. Ltd.) were used to produce images. Images were generated using a 40×objective, and were captured using Analysis FIVE software (Soft Imaging System Corp. 12596 W. Bayaud Ave. Suite 300 Lakewood CO 80228, USA) and analysed using Image Pro Plus 6.0 software (Media Cybernetics, Inc. 4340 East-West Hwy, Suite 400 Bethesda, MD 20814-4411 USA). Fibre type was identified based on staining characteristics [88]. Myotubes were defined as cells that appeared hollow in cross-section, the remainder were considered myofibres [9], [89]. Myofibres and myotubes were classified as type I (slow) myofibre, type I (slow) myotube, type II (fast) myofibre and type II (fast) myotube (**Figure S1**).

Morphological measurements were conducted by manually tracing anti-laminin-stained (rabbit anti-laminin, affinity isolated antibody: Sigma; diluted 1∶500 in PBS) margins of cells using the draw/merge object function of Image Pro Plus 6.0. For each fetus, the serial slow or fast stained myosin heavy chain slide with highest contrast was chosen to measure myofibre characteristics. Three fields (40× objective) of each chosen slide were analyzed. For each field, cross-sectional area (CSA) and number of type I (slow) myotubes and myofibres, type II (fast) myotubes and myofibres were measured. Furthermore, number and CSA were measured irrespective of cell type. All counted cells in the field comprised total cell number, and CSA of counted cells in the field was total cell CSA. For each myofibre characteristic an average was calculated of the three fields measured. For each fetus the average number of cells measured was 369, ranging from 152 to 705 cells. The average standard deviation between replicated fields for myofibre number was 1.3 for slow myotubes, 0.9 for slow myofibres, 5.1 for fast myotubes and 16.9 for fast myofibres. The average standard deviation between replicated fields for CSA was 43.3 µm^2^ for slow myotubes, 38.3 µm^2^ for slow myofibres, 19.7 µm^2^ for fast myotubes and 10.7 µm^2^ for fast myofibres.

### Expression of *H19* in Skeletal Muscle

Samples from *M. semitendinosus* were collected into RNA later (Qiagen, Chadstone Centre, VIC, Australia) immediately after recovery of fetuses in the abattoir and stored at −80°C after equilibration for 24 hours at 2–4°C. Total RNA was extracted from *M. semitendinosus* of all fetuses by TRI Reagent® Solution (Ambion, Life Technologies™ Inc., Carlsbad, CA, USA) according to the manufacturer’s instructions and RQ1-DNase treated (Promega, Madison, WI, USA). Reverse transcription was carried out using SuperScript™ III First-Strand synthesis system for RT-PCR (Invitrogen, Life Technologies™ Inc., Carlsbad, CA, USA) on 500 ng of total RNA with random hexamer oligonucleotides according to the manufacturer’s instructions. Amplification of *H19* from cDNA was performed using a forward primer located at the junction of exons 3 and 4, and a reverse primer located within exon 5 (**Table S6**). Total length of this amplicon was 171 bp. Real time quantitative PCR (qPCR) reactions were performed using Fast Start Universal SYBR Green Master (Roche Diagnostics GmbH, Mannheim, Germany) in an Eppendorf Mastercycler® pro S thermal cycler (Eppendorf Inc., Hamburg, Germany) on 4 µl of 40-fold diluted cDNA in a final volume of 12 µl with 6 µl of SYBR master mix (2×) at an annealing temperature of 60°C. Product specificity and integrity were confirmed using plots of melting curve and electrophoresis on a 2% agarose gel stained with GelRed™ Nucleic Acid Stain (Biotium Inc., Hayward, CA, USA). All qPCR experiments were performed in duplicate and the mean of both Cts used to calculate the amount of target transcript. We used the standard curve method with determination of PCR amplification efficiency. A two-fold serial dilution over eight data points was produced on a mixture of pooled cDNAs from all fetuses with equal proportions. Three replicates were used for each dilution of the cDNA template. Non-template control was included in all experiments. We determined relative expression levels of seven putative housekeeping genes including actin beta (*ACTB)*, ribosomal protein S9 (*RPS9*), ubiquitin B (*UBB*), H3 histone family 3A *(H3F3A)*, TATA box binding protein *(TBP)*, vacuolar protein sorting 4 homolog A (*VPS4A*) and cyclin G associated kinase (*GAK*) and used geNorm program version 3.5 [90] to identify *GAK* and *VPS4A* (see **Table S2**) as the most stable genes for normalization of the target gene. Expression levels of *H19* were normalized to the geometric mean of the expression levels of the selected housekeeping genes. As the normalized expression data were not normally distributed, we performed statistical analysis after logarithmic transformation of the data. The results for least square means and standard errors of means were presented after back-transformation.

### Statistical Estimation of Effects and Means

All data were analyzed by Univariate Analysis of Variance (ANOVA) using the general linear model (GLM) procedure of SPSS 17.0 (SPSS Inc., Chicago, IL, USA). Initially, data were fitted to the following full linear model:
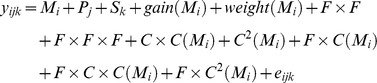
where *y_ijk_* were myofibre characteristics, muscle weights and transcript abundance, *M_i_* was maternal genome effect (*j* = Angus, Brahman), *P_j_* was paternal genome effect (*i* = Angus, Brahman), *S_k_* was fetal sex effect (*k* = male, female), *gain* was post-conception daily weight gain and *weight* was final maternal weight. *M_i_*, *P_j_* and *S_k_* were fitted as fixed factors (*F*) and *gain* and *weight* were fitted as covariates (*C*). The covariates fitted in the model were nested within maternal genome (*M_i_*) in order to adjust for effects of *gain* and *weight* within each of the two dam breeds. Interactions between factors and covariates were tested as follows: *F*×*F* was 2-way interaction between factors, *M_i_* ×*P_j_*, *M_i_*×*S_k_* and *P_j_*×*S_k_*, *F*×*F*×*F* was the 3-way interaction between factors, *M_i_*×*P_j_*×*S_k_*; *C*×*C*(*M_i_*) was the 2-way interaction of covariates nested within maternal genome, *gain*×weight(*M_i_*); *C^2^*(*M_i_*) was the quadratic term of covariates nested within maternal genetics, *gain* ^2^(*M_i_*) and *weight* ^2^(*M_i_*); *F*×*C*(*M_i_*) was the 2-way interaction between factors and covariates nested within maternal genetics, *P_j_*×*gain*(*M_i_*) and *S_k_*×*gain*(*M_i_*), *P_j_*×*weight*(*M_i_*) and *S_k_*×*weight*(*M_i_*); *F*×*C*×*C*(*M_i_*) was the 3-way interaction between factors and the two covariates nested within maternal genetics, *P_j_*×*gain*×*weight*(*M_i_*) and *S_k_*×*weight*×*gain*(*M_i_*); *F*×*C^2^* was the interaction between factors and quadratic terms of covariates nested within maternal genetics, *P_j_*×*gain* ^2^(*M_i_*), *S_k_*×*gain* ^2^(*M_i_*), *P_j_*×*weight* ^2^(*M_i_*) and *S_k_*×*weight* ^2^(*M_i_*).

Backward stepwise elimination was used to reduce the model for each measured parameter based on type III sums of squares (SSIII) at significance level (*P*) of 0.05. Type III sums of squares are independent of the order that effects are fitted in the model [91]. Specifically, elimination started with the least significant (largest *P* value) interaction or effect. Insignificant variables were removed stepwise according to marginality rules [92] i.e. independent variables cannot be eliminated until after the interaction is eliminated due to insignificance, and lower order interactions cannot be eliminated until after the corresponding higher order interaction is eliminated. Main effects were also considered to be marginalized by corresponding nested effects of covariates. Elimination continued until only significant effects and interactions remained, or had to be retained to maintain the marginality requirements. Main effects of *M_i_*, *P_j_* and *S_k_* were retained in the final model, irrespective of the significance levels. This approach retained factors of the experimental design and produced models with relatively large coefficients of determination (*R^2^*). *R* ^2^ values, model significance levels and significance levels of factors and nested covariates in the final model for each measured parameter are shown in **Table 1**. Means for effects of factors and interactions (with *P*-values from *t*-tests of the contrast, **Figures 3** **,** **4** **,** **6** **,** **7**) and regression slopes for nested effects of covariates (**Figure 5** **,** **7** and **Figure S3**) were plotted according to marginal means and estimated parameters obtained from the final model. *P*-values of maternal and/or paternal genome effects on fast myotube CSA, absolute weights of *M. supraspinatus, M. longissimus dorsi and M. quadriceps femoris*, and *H19* transcript abundance were not determined. The significant effects of final maternal weight nested within maternal genetics and/or significant interaction effects of maternal and paternal genome, would have biased *P*-values for corresponding main effects estimated with type III sums of squares (**Table1**, **Figure 3** **,** **4** **,** **7**).

Only one nested quadratic effect was significant when tested; *weight* ^2^(*M_i_*) explained a significant (*P* = 0.007) amount of variation in absolute *M.quadriceps femoris* weight. However, examination of plotted curves with individual data points revealed that this effect was dependent upon two heavy dams with high leverage. Therefore, this quadratic effect was removed from the model and the linear effect retained. The graph for the initial quadratic effect is presented in **Figure S3**.

The contribution of maternal genome (*M_i_*), paternal genome (*P_j_*), fetal sex (*S_k_*) and significant interaction and nested effects (*P*<0.05) to explained variation in myofibre characteristics, muscle weights and *H19* transcript abundance, was calculated from type I sums of squares (SSI). Type I sums of squares are dependent on the order in which effects are fitted in the model and sum to the total model SS [91], [92] (**Figures 1** **,** **2** **)**.

Final maternal weight (FMW) may contain both genetic and non-genetic effects as a function of breed and permanent environmental effect from origin of dam. Dams were sourced from different properties and had, therefore, been subject to different environments prior to recruitment for the experiment. By using SSI and fitting the maternal genome effect before *weight* in the model, we apportioned all the maternal genetic effect to maternal breed (*M_i_*) and left only environmental effects attributable to *weight*. Specifically, variables and/or interactions were fitted into the final SSI model in the following order:
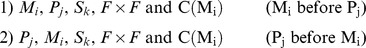



The SSI values of *P_j_* and *M_i_* were averaged from both models, assuming equal importance of maternal and paternal genomes. SSI values of other variables and interactions were identical for models 1 and 2. The SSI contribution of an interaction was apportioned equally to each component of the interaction. The contributions of maternal genetics (*M_i_*), paternal genetics (*P_j_*), fetal sex (*S_k_*) and final maternal weight (*weight*) to myofibre characteristics, muscle weights and transcript abundance were calculated from the SSI of *M_i_*, *P_j_*, *S_k_* and *weight* as a percentage of total SSI, respectively (**Figure 1**). The contribution of *weight* was defined as the non-genetic maternal effect, since the estimation of SSI values of *weight* were independent of maternal genome. The relative proportions of maternal and paternal genomes to total genetic variation in myofibre characteristics, muscle weights and transcript abundance were calculated by totalling respective contributions (**Figure 2**).

The regressions and Pearson correlation coefficients (*r*) for absolute and relative combined muscle weights and *H19* transcript abundance were estimated in SPSS 17.0 (SPSS Inc., Chicago, IL, USA).

## Supporting Information

Figure S1 **Example of immunohistochemical staining for fetal slow and fast myofibres in** ***M. semitendinosus*** **at midgestation.** (**A)** and (**B)** show serial stained sections of muscle tissue from one fetus against slow and fast myosin heavy chain isoforms, respectively. Arrows indicate slow myotubes (SMT), slow myofibres (SMF), fast myotubes (FMT) and fast myofibres (FMF).
(TIF)

Figure S2 **Fetal carcass weights for the four different combinations of maternal and paternal genomes and fetal sex at midgeststion.** Least square means with standard errors of means and *P*-values for significant differences (*t*-test) between means are indicated. Data were analyzed with a general linear model in SPSS 17.00 that included the factors fetal genetic group *i*, *i* = Bt×Bt, Bt×Bi, Bi×Bt, Bi×Bi (paternal genetics given first) and fetal sex *j*, *j* = male, female. The interaction between fetal genetic group and fetal sex was included in the model but removed as it was not significant (*P*>0.05).
(TIF)

Figure S3 **Quadratic effects of final maternal weight nested within maternal genomes on absolute weight of fetal** ***M. quadriceps femoris*** **at midgestation.** The *P*-value (ANOVA) of this nested effect is indicated. Bt: *Bos taurus taurus*, Angus. Bi: *Bos taurus indicus*, Brahman.
(TIF)

Figure S4 **Daily weight gain and final weight for** ***Bos taurus taurus*** **and** ***Bos taurus indicus*** **dams. (A)** Post-conception maternal daily gain: Final maternal weight – weight at conception divided by days of gestation. **(B)** Final maternal weight: Weight before slaughter on Day 153 of gestation. *P*-values for significantly different means (*t*-test) are indicated. Bt: *Bos taurus taurus*, Angus. Bi: *Bos taurus indicus*, Brahman.
(TIF)

Table S1 **Summary of distribution of maternal and paternal genomes and sex of fetuses.**
(DOCX)

Table S2 **Primer sequences used for quantitative real time polymerase chain reaction of** ***H19*** **and housekeeping genes.**
(DOCX)
