# Supplementary figures and images for: Maternal and Paternal Genomes Differentially Affect Myofibre Characteristics and Muscle Weights of Bovine Fetuses at Midgestation
Source: PLoS One. 2013 Jan 14;8(1):e53402. doi: 10.1371/journal.pone.0053402 (PMC3544898; doi:10.1371/journal.pone.0053402)

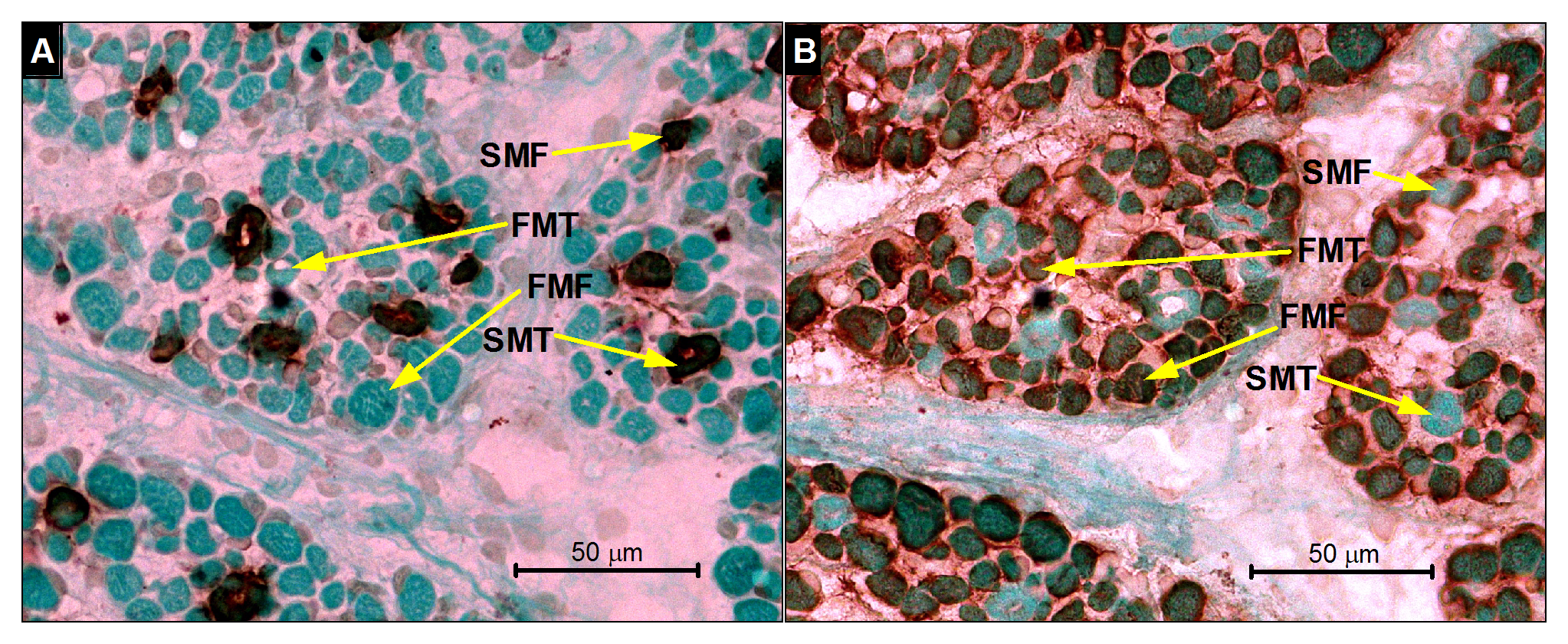

Supplement: Figure S1 — Example of immunohistochemical staining for fetal slow and fast myofibres in M. semitendinosus at midgestation. (A) and (B) show serial stained sections of muscle tissue from one fetus against slow and fast myosin heavy chain isoforms, respectively. Arrows indicate slow myotubes (SMT), slow myofibres (SMF), fast myotubes (FMT) and fast myofibres (FMF). (TIF) [file pone.0053402.s001.tif]

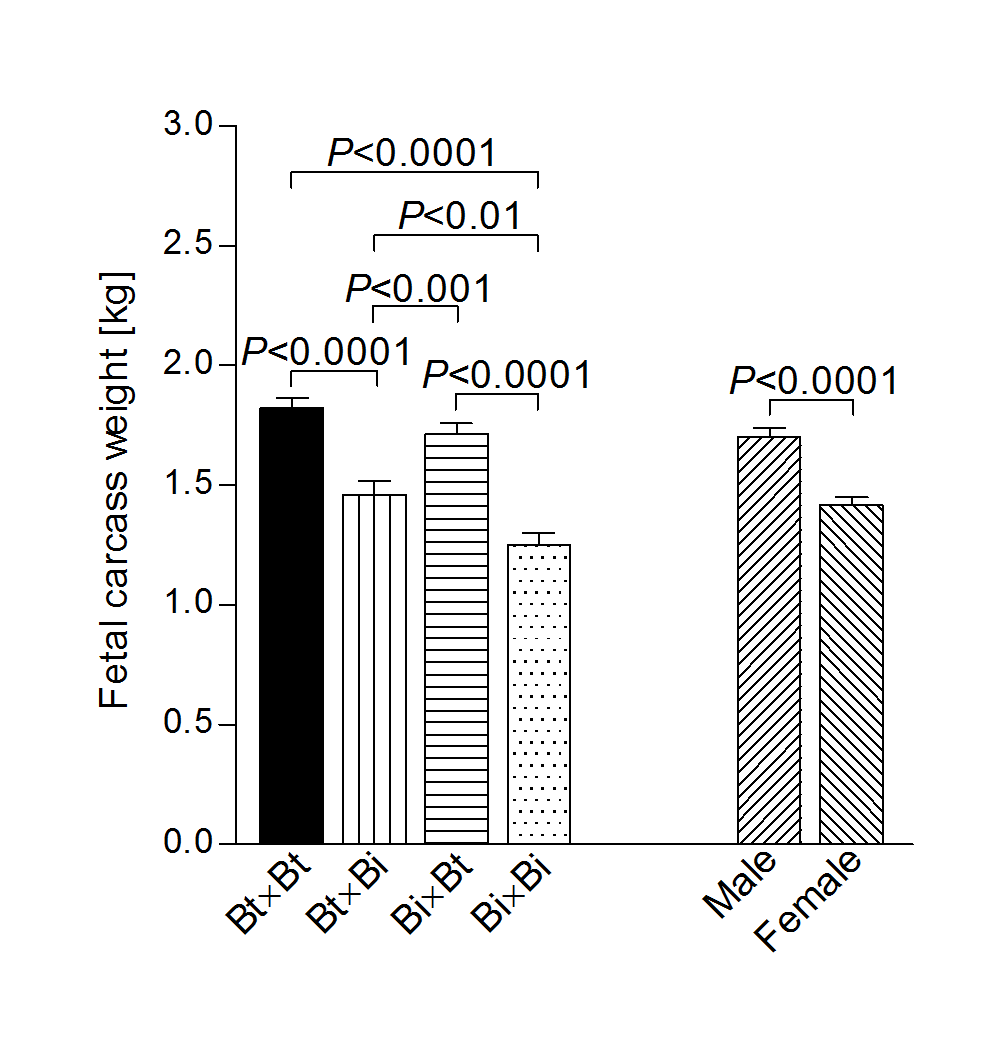

Supplement: Figure S2 — Fetal carcass weights for the four different combinations of maternal and paternal genomes and fetal sex at midgeststion. Least square means with standard errors of means and P-values for significant differences (t-test) between means are indicated. Data were analyzed with a general linear model in SPSS 17.00 that included the factors fetal genetic group i, i = Bt×Bt, Bt×Bi, Bi×Bt, Bi×Bi (paternal genetics given first) and fetal sex j, j = male, female. The interaction between fetal genetic group and fetal sex was included in the model but removed as it was not significant (P>0.05). (TIF) [file pone.0053402.s002.tif]

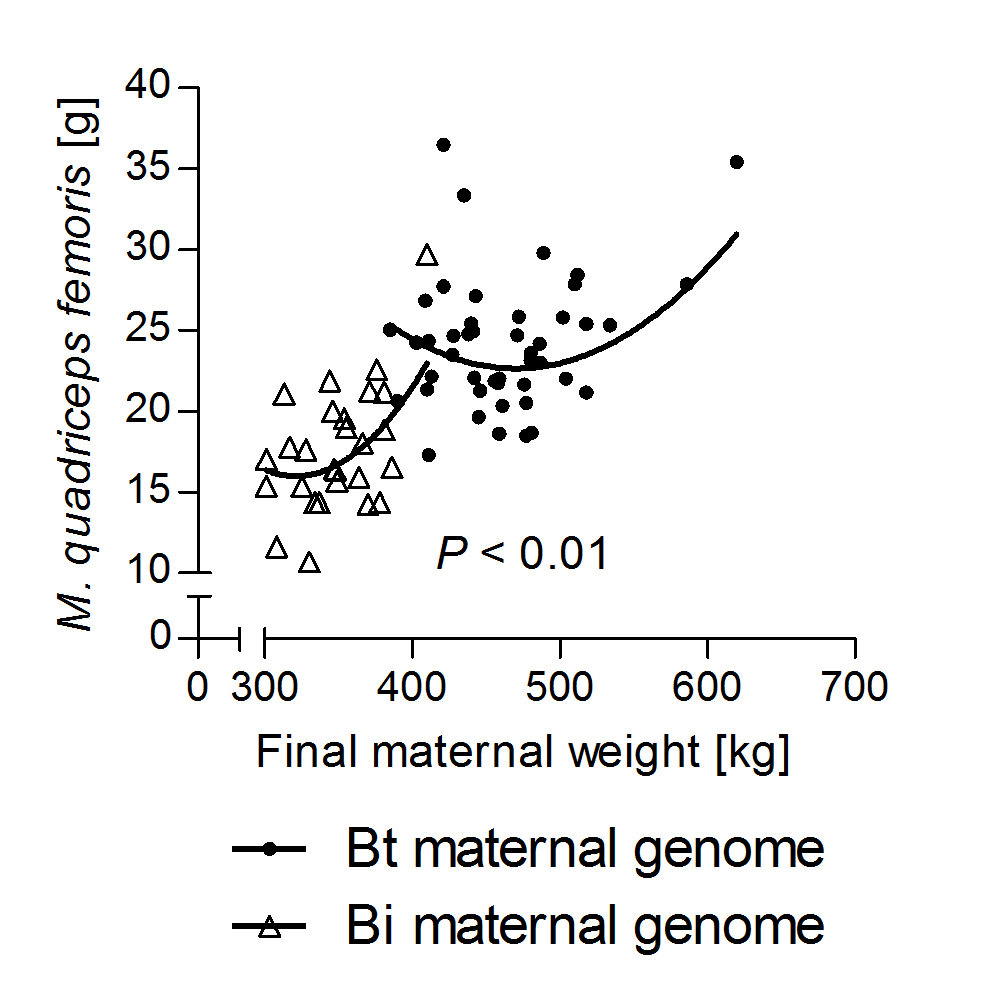

Supplement: Figure S3 — Quadratic effects of final maternal weight nested within maternal genomes on absolute weight of fetal M. quadriceps femoris at midgestation. The P-value (ANOVA) of this nested effect is indicated. Bt: Bos taurus taurus, Angus. Bi: Bos taurus indicus, Brahman. (TIF) [file pone.0053402.s003.tif]

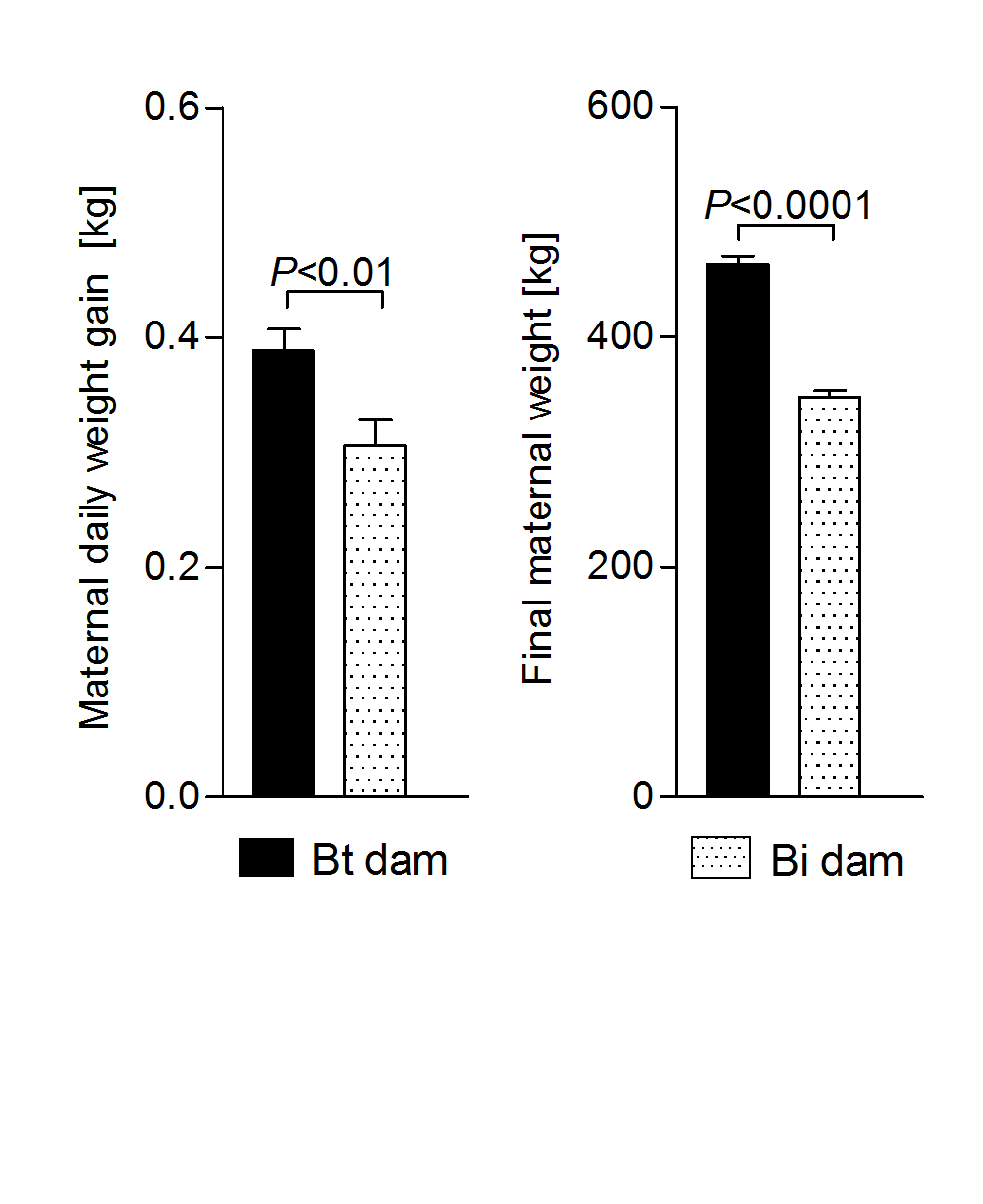

Supplement: Figure S4 — Daily weight gain and final weight for Bos taurus taurus and Bos taurus indicus dams. (A) Post-conception maternal daily gain: Final maternal weight – weight at conception divided by days of gestation. (B) Final maternal weight: Weight before slaughter on Day 153 of gestation. P-values for significantly different means (t-test) are indicated. Bt: Bos taurus taurus, Angus. Bi: Bos taurus indicus, Brahman. (TIF) [file pone.0053402.s004.tif]
