# Supplementary material for: Maternal and Paternal Genomes Differentially Affect Myofibre Characteristics and Muscle Weights of Bovine Fetuses at Midgestation
Source: PLoS One. 2013 Jan 14;8(1):e53402. doi: 10.1371/journal.pone.0053402 (PMC3544898; doi:10.1371/journal.pone.0053402)
Supplement: Table S1 — Summary of distribution of maternal and paternal genomes and sex of fetuses. (DOCX) [file pone.0053402.s005.docx]

| **Table S1.** Summary for distribution of maternal and paternal genomes and sex of fetuses | | |
| --- | --- | --- |
|  |  | n |
| Maternal genome | Angus | 45 |
|  | Brahman | 28 |
| Paternal genome | Angus | 36 |
|  | Brahman | 37 |
| Fetal sex | Male | 27 |
|  | Female | 46 |
