# Supplementary material for: Maternal and Paternal Genomes Differentially Affect Myofibre Characteristics and Muscle Weights of Bovine Fetuses at Midgestation
Source: PLoS One. 2013 Jan 14;8(1):e53402. doi: 10.1371/journal.pone.0053402 (PMC3544898; doi:10.1371/journal.pone.0053402)
Supplement: Table S2 — Primer sequences used for quantitative real time polymerase chain reaction of H19 and housekeeping genes. (DOCX) [file pone.0053402.s006.docx]

| **Table S2.** Primer sequences used for quantitative real time polymerase chain reaction of *H19* and housekeeping genes | | | |
| --- | --- | --- | --- |
| Primer name | Sequence (5’ to 3’) | Annealing temperature | Fragment size |
| H19-F | TCAAGATGACAAGAGATGGTGCTA | 60 ºC | 171 bp |
| H19-R | GGTGTGGGTCGTCCGTTC | 60 ºC | 171 bp |
| VPS4A-F | GAAGACAGAAGGCTACTCGGGTG | 60 ºC | 106 bp |
| VPS4A-R | ACAGACCTTTTTGAAGTGTGTTGCT | 60 ºC | 106 bp |
| GAK-F | CACGACCATCTCACACTACCCA | 60 ºC | 128 bp |
| GAK-R | AGTTTGAGTACAAGTCCACAATTTCC | 60 ºC | 128 bp |
|  |  |  |  |
